# Hyponatremia and other potential markers of ultrasound abnormalities after a first febrile urinary tract infection in children

**DOI:** 10.1007/s00431-023-05149-z

**Published:** 2023-08-17

**Authors:** Isabel González-Bertolín, Guillermo Barbas Bernardos, Leire García Suarez, Rosario López López, Paula García Sánchez, Patricia Bote Gascón, Cristina Calvo

**Affiliations:** 1 Pediatric Emergency Department, La Paz University Hospital, Madrid, Spain; 2 Urology Department, Clínica Universidad de Navarra, Marquesado de Santa Marta, 1, 28027 Madrid, Spain; 3 Pediatric Nephrology Department, La Paz University Hospital, Madrid, Spain; 4 Pediatric Nephrology Department, Fuerteventura Virgen de la Peña General Hospital, Fuerteventura, Spain; 5 Pediatrics and Infectious Disease Department, La Paz University Hospital, IdiPaz Foundation. Traslational Research Network in Pediatric Infectious Diseases (RITIP), CIBERINFEC. ISCIII, Madrid, Spain

**Keywords:** Hyponatremia, Febrile urinary tract infection, Urinary tract malformations, Marker

## Abstract

Urinary tract infections are the initial manifestation in 30% of urinary tract malformations. Identifying these patients, who could benefit from a specific treatment, is still challenging. Hyponatremia during urinary tract infection has been proposed as a urinary tract malformation marker. We evaluate the prevalence of hyponatremia during febrile urinary tract infections and its association with subjacent urinary tract malformations. We performed a retrospective study of healthy patients under 16 years, diagnosed with a first episode of febrile urinary tract infection, who had undergone blood testing in the acute episode and at least one renal ultrasound during follow-up (January 2014-November 2020). Hyponatremia was defined as (serum sodium ≤ 130 mEq/L). According to imaging findings, we classified patients into three groups: normal kidney ultrasound, mild pelviectasis, and significant urinary tract malformation. We performed logistic regression models to identify independent risk factors for urinary tract malformation and mild pelviectasis. We included 492 patients and 2.8% presented hyponatremia. We identified normal ultrasound in 77%, mild pelviectasis in 10.8%, and urinary tract malformation in 12% of patients. We found an association between mild pelviectasis and hyponatremia [OR 6.6 (CI95% 1.6–26.6)]. However, we found no association between hyponatremia and urinary tract malformation. The parameters that were associated with malformations were presenting a non-*E. coli* infection, C-reactive-protein levels over 80 mg/L, and bacteremia.

*Conclusion*: Hyponatremia during the first episode of febrile urinary tract infection is present in 2.8% of patients and is associated with mild pelviectasis in imaging. However, hyponatremia does not indicate a greater need for complementary tests to screen for urinary tract malformations.

**Table:** 

**What is Known:** *• Urinary tract infection is the first manifestation in 30% of children with urinary tract malformation.* *• Hyponatremia could be a marker to identify these children and guide the imaging approach.*	
**What is New:** *• Around 12% of children with a first episode of febrile urinary tract infection have a urinary tract malformation.* *• Non-E. coli infection, C-reactive protein levels over 80 mg/L, and bacteremia are markers for malformations to guide diagnostic imaging tests, but hyponatremia (Na ≤ 130 mEq/l) is not a reliable marker.*

**Supplementary Information:**

The online version contains supplementary material available at 10.1007/s00431-023-05149-z.

## Background

Urinary tract infections (UTIs) are the first clinical manifestation of urinary tract malformations in up to 30% of the patients suffering from them [1]. Identifying patients who require targeted treatment remains controversial [2–4].

Clinical guidelines lack consensus on the recommended work-up after a first febrile UTI. Renal ultrasound is variably recommended for all patients to infants under six months without alarming signs.

Voiding cystourethrogram (VCUG), an invasive radiating technique, should be tailored based on individual risk. Different guidelines consider renal ultrasound abnormalities and additional risk factors for recommending VCUG [5]. The European Association of Urology/European Society for Paediatric Urology suggests VCUG screening for vesicoureteral reflux (VUR) in all infants with febrile UTI. Amid this controversy, various markers have been proposed to identify patients for complementary testing, including hyponatremia [4, 6]. Hyponatremia is common in adults, particularly those with chronic diseases or certain medications, and in infants and children with conditions like gastroenteritis or bronchiolitis [7, 8]. Up to two-thirds of children with febrile UTIs may have low serum sodium levels, more frequent than other pathologies causing fever [9]. Studies have linked hyponatremia during UTI to urinary tract malformations, suggesting screening in the presence of this electrolyte imbalance [6, 10–12]. However, it is still controversial whether this analytic finding is more frequent in patients with urinary tract malformations or if it appears similarly in patients with febrile UTI without nephro-urological subjacent conditions [7, 9, 13, 14]. This study aims to assess hyponatremia prevalence in previously healthy children with a first febrile UTI and its association with previously undetected malformations. The study also aims to identify independent risk factors for urinary tract malformations in febrile UTI cases.

## Patients and methods

### Study design

We performed an observational retrospective study including all patients under 16 years old diagnosed with febrile UTI in a tertiary hospital's ED from January 1st, 2014, to November 30th, 2020.

### Population/patients

#### Inclusion criteria

1) Patients who met diagnostic criteria for febrile UTI, 2) patients aged less than 16 years, 3) patients who underwent a blood test including sodium measurement at ED admission, 4) patients undergoing at least one renal ultrasound during follow-up with a minimum overall study follow-up of 2 years.

#### Exclusion criteria

1) patients with a previous medical history of UTI, 2) previously known urinary tract malformation of any kind, 3) kidney transplant recipients, 4) previous kidney function impairment, 5) immunodeficient patients, 6) chronic medical conditions (including multiresistant bacterial colonization), and 7) patients already diagnosed with UTI under treatment.

### Variables

The variables analyzed were: sex, age, serum C-reactive protein (CRP) levels, serum sodium concentration, serum creatinine concentration, blood leukocyte and neutrophil count, duration of fever, maximum registered temperature (referred by parents or recorded in the ED), presence of vomiting, clinical dehydration, non-*E. Coli* infection detected in urine culture and positivity of blood culture. Renal ultrasound findings in all patients and VCUG findings, when performed, were also registered for analysis.

In our hospital, clinical dehydration is systematically documented as well-hydrated or dehydrated in each patient's medical records. Clinicians evaluate the presence of dehydration based on objective and subjective criteria, taking into account signs and symptoms such as decreased skin turgor, dry mucous membranes, sunken fontanelle, decreased urine output, irritability, and abnormal vital signs. However, no clinical dehydration scale is systematically used by all pediatricians. One mild symptom, such as dry buccal mucous membranes, is normally sufficient for pediatric attendants to classify a child as dehydrated.

The samples in our study were analyzed by indirect potentiometry for sodium measurement (Atellica^®^ Solution Immunoassay & Clinical Chemistry Analyzers, Siemens, Germany), commonly used in clinical laboratories. However, we also utilized direct potentiometry (ABL 90 flex^®^ gas analyzer, Radiometer, Denmark) to ensure accuracy when the portion of serum occupied by lipids and proteins differed from the typical value of 7%. This approach allowed us to consider the potential impact of protein and lipid concentrations on sodium measurement and validate our results [15].

### Definitions

- Febrile UTI: Temperature over 38 ºC and a compatible urinalysis: significant leukocyturia or nitrituria and culture-proven bacteriuria in collected sterile urine (> 10.000 CFU/ml in transurethral bladder catheterization, > 1.000 CFU/ml in suprapubic bladder aspiration or > 100.000 CFU/ml in clean-catch urine collection).
- Normonatremia: serum sodium concentration 135–145 mEq/L.
- Clinically relevant hyponatremia: serum sodium concentration ≤ 130 mEq/L (moderate: 130–121 mEq/L, severe: ≤ 120 mEq/L).
- Acute kidney injury (AKI): creatinine elevation ≥ 1.5 the median for age. The 2012 KDIGO classification [16] of acute kidney injury includes in its definitions the basal creatinine, often unknown in the pediatric population with no previous medical conditions in need of blood sampling. Therefore, our study used median values for each age group established by Pottel et al. [17] as reference creatinine levels.

### Imaging classification

We classified imaging findings into three groups according to the Urinary tract dilatation (UTD) classification system [18] and the definitions of the International Grading System [19] to evaluate VUR:

- Normal
- Mild pelviectasis with no other abnormalities: Urinary tract dilatation = P1. It was defined as a normal urinary tract with an anterior–posterior renal pelvic diameter of 10 to < 15 mm and central calyceal dilation.
- Significant urinary tract malformation: Urinary tract dilatation > P1. Anterior–posterior renal pelvic diameter ≥ 15 mm or peripheral calyceal dilation, additional ureteral dilation, abnormal renal echogenicity or cysts, or bladder abnormalities, regardless of anterior–posterior renal pelvic diameter measurement. VUR.

### Study protocol

Our Institution protocol includes the performance of blood tests in the ED for every patient with a suspected febrile UTI and an ambulatory renal ultrasound after a microbiologically confirmed febrile UTI. If ultrasound abnormalities compatible with VUR or pyelonephritis are detected, or in case of recurrent UTI, VCUG is performed for VUR screening. Experienced pediatric radiologists performed all imaging tests.

### Statistical analysis

Data was informatically processed with a Microsoft Excel file imported for its statistical analysis in SAS version 9.4 (SAS Institute Inc. 2013. Base SAS^®^ 9.4 SAS/STAT – Statistical analysis. Cary, NC). Statistically significance was defined as a probability of error lower than 5% (*p* < 0.05).

In the first stage, we performed a statistical analysis comparing the three renal ultrasound groups (normal, mild pylectasia, and significant urinary tract malformation) using the ANOVA test for continuous parametric quantitative variables and the Kruskal-Wallis test for non-parametric. The frequency analysis between qualitative variables was performed using Yates’s Chi-squared test. We applied Bonferroni’s correction to avoid multiple comparison bias. We performed a complete case analysis, excluding cases with missing values on any variable of interest.

After simple statistical analysis, a multivariate analysis using logistic regression was performed to identify factors with an independent effect on the presence of urinary tract malformations and mild pelviectasis. In this analysis statistical significance variables, those with a trend towards statistical significance (*p* < 0.1) in the simple initial analysis, and those with a recognized clinical relevance were included.

## Results

**Fig. 1:**
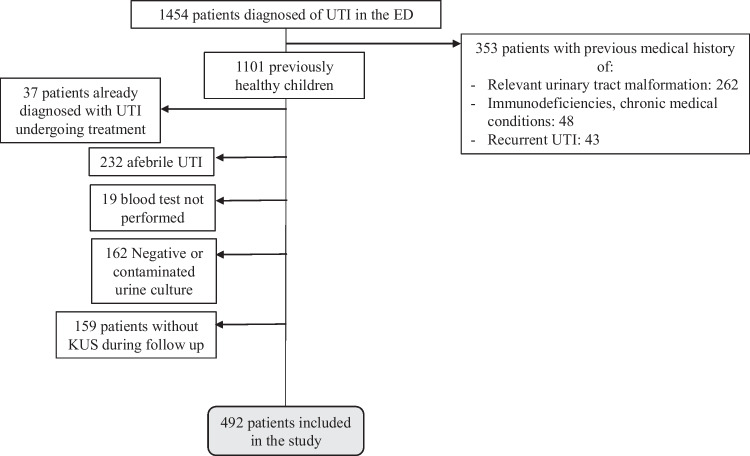
Flow diagram to indicate the included and excluded patients

A total of 1454 patients were diagnosed with febrile UTI in the ED during the inclusion period, of whom 492 met the inclusion criteria Fig. 1.

Thirty-eight percent were males (189/492) with a median age of seven months (p25-p75: 3–14). The most frequently isolated pathogen in urine culture was *Escherichia coli* (95%). Other bacteria implicated were: *Proteus spp* (2.6%), *Klebsiella spp* (1.3%), *Enterococcus spp* (0.7%), and *Citrobacter spp* (0.1%). From 450 blood cultures performed, 20 (4.4%) were positive, all with *E. coli* isolation. A coagulase-negative *Staphylococcus* was implicated in 15 (3.3%), considering this result a contaminated sample.

Hyponatremia, defined as serum sodium concentration ≤ 130 mEq/L, was present in 14 patients (2.8%), and no severe hyponatremias were detected.

All patients underwent renal ultrasound within a median of 9 days (p25-75:1–28) after the febrile UTI episode. Of the 492 patients, 381 (77%) did not have any renal ultrasound abnormalities, and 111 (23%) presented with some alteration. Within the group of abnormal imaging findings, 53/111 (10.8% of the total sample) had mild pelviectasis (UTD P1), and 58/111 (12% of the full sample) had significant urinary tract malformation (Table 1).

**Table 1:** Frequency of Urinary Tract Malformation Types

**URINARY TRACT MALFORMATION**		***N*** **= 58**
**Urinary tract dilatation**		19 (33)
**Vesicoureteral reflux**		30 (52)
	grade I	0 (0)
	grade II	14 (24)
	grade III	10 (17)
	grade IV	6 (10)
	grade V	0 (0)
**Renal duplicity**		9 (15)
**Parenchymal malformation**		0 (0)

The table provides the frequency of different types of urinary tract malformations. Data are presented as absolute frequencies with the percentage in parentheses. Urinary tract dilatation was classified according to the Urinary Tract Dilatation Classification System, including children with a grade greater than P1, which encompasses abnormalities such as anterior–posterior renal pelvic diameter ≥ 15 mm or peripheral calyceal dilation, additional ureteral dilation, abnormal renal echogenicity or cysts, or bladder abnormalities, regardless of anterior–posterior renal pelvic diameter measurement. Vesicoureteral reflux was classified according to the International Grading System

According to image findings, patients were classified into the previously mentioned three groups: normal ultrasound, mild pelviectasis, and significant urinary tract malformation. Clinical, demographic characteristics, and analytic blood results in each group are presented in Table 2. There were no significant differences in age, sex, presence of vomiting, clinical dehydration symptoms, or fever duration. However, differences were found within the three groups in moderate hyponatremia, with 9.4% of the patients in the mild pelviectasis group, 5.2% in the urinary tract malformation, and 1.6% in the normal ultrasound group being hyponatremic (*p* = 0.003).

**Table 2:** Patients’ demographic, clinical and laboratory data

	**Normal** **ultrasound** (*n* = 381)	**Mild** **pelviectasis** (*n* = 53)	**Urinary** **Tract** **malformation** (*n* = 58)	***P* values**
	*Median (p25-p75)*			
**Age (months)**	7 (3–13)	10 (4–21)	5 (2–22)	0.13
**Fever (hours)**	24 (12–72)	48 (12–96)	24 (7–48)	0.09

	*Mean (SD)*			
**Temperature (ºC)**	39.1 (0.8)	39.4 (0.7)	39.1 (0.8)	**0.03**
**Leukocytes (/mm**^**3**^**)**	17890 (8784)	19609 (6675)	18132 (6596)	0.22
**Neutrophils (/mm**^**3**^**)**	10056 (5319)	12072 (5316)	10943 (5005)	**0.026**
**CRP value (mg/l)**	87.2 (75)	125 (83.2)	98.7 (73)	**0.003**

	*Absolute frequencies (%)*			
**Sex (male)**	149 (39)	17 (32)	123 (40)	0.2
**Hyponatremia**	6 (1.6)	5 (9.4)	3 (5.2)	**0.003**
**Vomits**	128 (34)	20 (38)	19 (33)	0.82
**Dehydration**	7 (1.8)	1 (1.9)	1 (1.7)	0.99
**Acute kidney injury**	50 (13)	13 (25)	9 (16)	0.09
**Bacteremia**	12 (3.5)	2 (4)	6 (12)	**0.032**
**Non-*E.coli* infection**	14 (3.7)	3 (5.7)	6 (10.3)	0.07

Data are presented as the median (mdn), with the interquartile range (p25-p75) in the parentheses, as the mean (m) with the standard deviation (SD) in the parentheses, or as absolute frequencies (AF) with the percentage in parentheses

*CRP* C-Reactive protein

Bold data represent statistically significant values (*p*<0.05)

Multivariate analysis comparing mild pelviectasis and urinary tract malformation groups with normal ultrasound demonstrated that hyponatremia did not have a statistically significant correlation with urinary tract malformations but represented an independent risk factor for mild pelviectasis [OR 6.6 (CI95% 1.6 - 26.6]. CRP levels over 80 mg/L also represented an independent risk factor for this renal ultrasound finding [OR 2.6 (CI95% 1.64- 4.9)]. Furthermore, AKI was also associated with mild pelviectasis [OR 2.3 (CI95% 1.1- 4.9)].

**Fig. 2:**
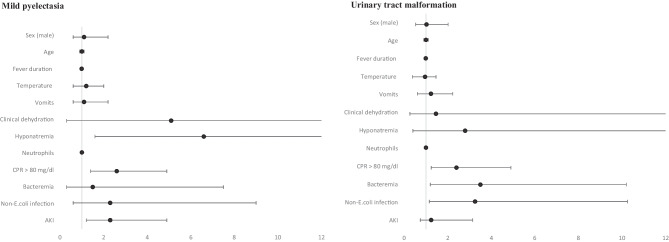
Multivariate analysis of risk for presenting mild pelviectasis and urinary tract malformation in imaging test. Footnote: AKI: acute kidney injury. CRP: C-Reactive protein

On the other hand, parameters identified as independent risk factors for urinary tract malformations were: non-*E. coli* infection [OR 3.25 (CI95% 1.04- 10.2)], CRP levels over 80 mg/L [OR 2.4 (CI95% 1.16- 4.9)] and having a positive blood culture (OR 3.49, CI95% 1.18 – 10.3) Fig. 2. Additional information, including detailed data tables, is provided in the Supplementary Material (available online).

## Discussion

Our study found that 12% of previously healthy children with their first febrile urinary tract infection (UTI) had an unknown clinically relevant urinary tract malformation. Furthermore, 2.8% of these children had hyponatremia, which did not correlate with a urinary tract malformation but was associated with mild pelviectasis. Independent risk factors for urinary tract malformation were non-*E. coli* infection, CRP levels over 80 mg/L, and bacteremia. Independent risk factors for mild pelviectasis were hyponatremia, CRP > 80 mg/L, and acute kidney injury (AKI) at the moment of febrile UTI diagnosis.

Clinically relevant hyponatremia, defined as a serum sodium concentration ≤ 130 mEq/L, was present in 2.8% of children with febrile UTI, all of whom had moderate hyponatremia. These findings are similar to a study by Pappo et al. [7], which observed a prevalence of 3.9% for moderate hyponatremia in hospitalized patients. Their study, which included 233 patients with ages similar to those in our cohort, did not identify any cases of severe hyponatremia. The numbers obtained in their work are slightly higher than ours, probably because they only included hospitalized patients who presumably are at higher risk of hyponatremia. Additionally, the sex distribution in their study (32% male) aligns with the 38% male distribution observed in our series.

The pathophysiological mechanism of hyponatremia in UTI patients is still unclear and may involve factors such as vomiting, low intake, increased fluid losses, aldosterone resistance, and inadequate antidiuretic hormone secretion [6, 14]. Inflammatory cytokines and urinary tract dilation have also been implicated in the development of hyponatremia [8, 20].

It has been suggested that patients with urinary tract malformations are at a higher risk of pseudohypoaldosteronism-mediated hyponatremia in the context of a UTI. However, most previous studies have involved small populations [12, 20]. Our study demonstrates that clinically relevant hyponatremia is an independent risk factor for renal ultrasound abnormalities. However, as shown in other studies [13, 21], we did not find an association between hyponatremia and severe or significant urinary tract malformations.

In our series, we identified an association between mild pelviectasis and hyponatremia. Additionally, serum CRP levels and AKI were also associated with mild pelviectasis. Both hyponatremia and elevated CRP levels have been linked to renal parenchymal inflammation during acute pyelonephritis [14]. Previous studies have shown a correlation between gammagraphic abnormalities and hyponatremia and an association between lower serum sodium concentration, higher CRP levels, and more severe illness in UTI patients [7, 14].

AKI in the pediatric population with UTI has been scarcely studied. Studies in adults suggest that it could be considered a marker of pyelonephritis vs. cystitis since upper UTI increases 2.63 times (CI95% 1.53–4.56) the risk of AKI [22]. In our work, as reference creatinine the median values for each age group established by Pottel et al. [17] were used. According to our cohort, the prevalence of AKI during a febrile UTI is 15%, and the increase in serum creatinine 1.5 times the median for age entailed a 2.3 times (CI95% 1.1–4.9) higher risk of finding mild pelviectasis in the renal ultrasound.

Similarly to these three parameters; hyponatremia, CRP, and AKI; the detected association with mild pelviectasis may reflect inflammation of the kidney parenchyma. In pyelonephritis, obstruction to normal urine flow can be found due to two mechanisms: peristalsis inhibition secondary to bacterial endotoxins that block α-adrenergic receptors within the smooth muscle, thus creating a functional obstruction, and the proper urothelium inflammation known as ureteropyelitis [23]. These two mechanisms can produce the onset of mild pelviectasis and occasionally can be demonstrated radiologically before renal parenchymal changes evolve [24]. These changes are believed to be reversible after the acute process is resolved [23]. Mild pelviectasis has even been considered in some papers as a renal ultrasound finding compatible with acute pyelonephritis [23, 24]. Our study is the first to describe its relation with inflammatory parameters, unlike structural malformations.

In addition, relevant urinary tract malformations were associated with different parameters in our study. Non-*E. coli* positive urine culture is an excellent subjacent urinary tract malformation marker, increasing the risk by almost 4 times. This finding aligns with previous studies and guidelines recommending VUR screening in patients with a first febrile urinary tract infection (UTI) caused by non-*E. coli* microorganisms.

Another less studied parameter, significantly associated with urinary tract malformations, is the positivity of blood cultures. Up to 4% of our patients had a bacteremia. Hoberman et al. reported the same prevalence in their study of 306 children [25]. To the best of our knowledge, only the paper by Honkinen et al. [26] had previously documented this association, demonstrating a higher prevalence of obstructive uropathy (9%) in 135 patients with febrile UTI and bacteremia versus 1% of patients with non-bacteremic febrile UTI (*p* < 0.01). These findings contrast sharply with ours, in which 33% of patients with bacteremia and 9% of the negative blood cultures presented with some urinary tract malformations (*p* = 0.024). This difference may be justified by the fact that different types of malformation and not only obstructive uropathy were considered.

Increased CRP levels during acute UTI have been related to gammagraphic defects in DMSA, indicating parenchymal damage, which correlates with urinary tract malformations such as VUR [5]. Regarding our results, CRP > 80 mg/dl is associated with a 2.6-fold increased risk of urinary tract malformation.

Our findings support the hypothesis that low serum sodium concentration represents an inflammatory marker but not a subjacent urinary tract malformation marker.

Our study has some limitations. First, the retrospective study design may have resulted in the loss of information due to incomplete clinical reports. Secondly, the decision to perform additional tests, such as VCUG or DMSA, relied on medical criteria, leading to potential variability in clinical recommendations and management. However, as this was a monocentric study, the use of well-defined protocols by all staff members may have minimized this bias. Additionally, limited access to data on prenatal ultrasounds in all clinical reports may have impacted the analysis. However, most women in our country are believed to undergo adequate monitoring during pregnancy. Furthermore, the fact that renal ultrasound represents a diagnostic technique with a high inter-observer variability may have also influenced the results. However, it must be highlighted that experienced pediatric radiologists performed all renal ultrasound, which is expected to have tempered this limitation.

The method for calculating AKI may be subject to bias, given that creatinine is related to the individual's muscle mass. Thus, AKI in undernourished or short patients may be underestimated, while in those with high weight for age or tall patients, it may be overestimated [27]. However, these reference values according to age are often the only available data in daily clinical practice in EDs to estimate the deterioration of kidney function.

Another limitation is the use of the indirect potentiometry method for sodium measurement. However, this was partially addressed by incorporating direct potentiometry when the serum composition differed from the typical value of 7% occupied by lipids and proteins [15, 28]. Finally, due to the study characteristics, serial renal ultrasound to monitor the radiologic findings' evolution over time could not be accessed. In the absence of specific conditions (atypical germs, persistent fever of more than 72 h, etc.), guidelines recommendations vary on the timing of renal ultrasound after a UTI in children from 24 h to 6 weeks after the episode. The median of 9 days (p25-p75: 1–28) to perform a renal ultrasound after a urinary tract infection (UTI) in our series falls within the suggested timing range. However, it would be of great interest to perform prospective studies that evaluate radiologic changes over time to shed some light on whether these findings in the acute moment are reversible. If our hypothesis was true, complementary tests, such as VCUG, could be avoided in patients whose ultrasound abnormalities would resolve.

This study's main strengths rely on the large number of patients involved, our stringent definition of hyponatremia (sodium ≤ 130 mmol/L) which could require specific treatment or could have more relevant prognostic implications than 135 mmol/L cut-off, and the utilization of a multivariate analysis approach, allowing for a comprehensive evaluation of independent risk factors and their association with urinary tract malformations and mild pelviectasis.

As a summary, we found that one in 10 children presenting in the ED with a first febrile UTI have an underlying urinary tract malformation, and around 3% suffered hyponatremia with a serum sodium concentration ≤ 130 mEq/L. According to our results, this analytic finding cannot be considered a good urinary tract malformation marker. Still, it can be considered an independent marker of mild pelviectasis which could reflect kidney parenchyma and urinary tract / epithelial inflammation during the acute phase of the disease.

Independent risk factors for urinary tract malformations identified in our study were non-*E. coli* infection, CRP levels over 80 mg/L, and bacteremia.

### Supplementary Information

Below is the link to the electronic supplementary material.

Supplementary file1 (DOCX 19 kb)

## List of abbreviations

AKI: Acute kidney injury
CRP: C-reactive protein
DMSA: Dimercaptosuccinic acid
ED: Emergency Department
UTI: Urinary tract infection
VCUG: Voiding cystourethrogram
VUR: Vesicoureteral reflux
