# Supplementary material for: Hyponatremia and other potential markers of ultrasound abnormalities after a first febrile urinary tract infection in children
Source: Eur J Pediatr. 2023 Aug 17;182(11):4867–74. doi: 10.1007/s00431-023-05149-z (PMC10640435; doi:10.1007/s00431-023-05149-z)
Supplement: Supplementary file 1 — Supplementary file1 (DOCX 19 kb) [file 431_2023_5149_MOESM1_ESM.docx]

|  | Sig. | Exp(B) | CI95% Inferior | CI95% Superior |
| --- | --- | --- | --- | --- |
| Sex (male) | 0.941 | 1.025 | 0.529 | 1.985 |
| Age | 0.902 | 1.001 | 0.988 | 1.013 |
| Fever duration | 0.127 | 0.993 | 0.985 | 1.002 |
| Temperature | 0.862 | 0.957 | 0.584 | 1.567 |
| Vomits | 0.559 | 1.228 | 0.617 | 2.441 |
| Clinical dehydration | 0.754 | 1.464 | 0.135 | 15.872 |
| Hyponatremia | 0.261 | 2.847 | 0.459 | 17.667 |
| Neutrophils | 0.831 | 1.000 | 1.000 | 1.000 |
| CRP >80 mg/dl | 0.018 | 2.406 | 1.166 | 4.963 |
| Bacteremia | 0.024 | 3.488 | 1.183 | 10.284 |
| Non*-E. coli* infection | 0.043 | 3.255 | 1.040 | 10.187 |
| Acute Kidney Injury | 0.636 | 1.248 | 0.498 | 3.126 |
| Constant | 0.913 | 0.343 |  |  |

**Supplementary material**

Multivariate analysis of risk for presenting mild pelviectasis in imaging test

Note: The dependent variable in the logistic regression model is the is the presence of mild pelviectasis (vs. absence of imaging abnormalities). The table presents the independent variables included in the analysis and their corresponding statistical associations with mild pelviectasis. expressed as odds ratios [Exp(B)] with 95% confidence intervals (CI) and significance value (Sig.). CRP: C-Reactive protein.

Multivariate analysis of risk for presenting urinary tract malformation in imaging test

|  | Sig. | Exp(B) | CI95% Inferior | CI95% Superior |
| --- | --- | --- | --- | --- |
| Sex (male) | 0.072 | 1.111 | 0.546 | 2.260 |
| Age | 0.962 | 1.000 | 0.988 | 1.011 |
| Fever duration | 0.977 | 1.000 | 0.993 | 1.007 |
| Temperature | 0.573 | 1.159 | 0.693 | 1.938 |
| Vomits | 0.722 | 1.132 | 0.570 | 2.248 |
| Clinical dehydration | 0.225 | 5.114 | 0.366 | 71.499 |
| Hyponatremia | 0.008 | 6.601 | 1.598 | 26.610 |
| Neutrophils | 0.429 | 1.000 | 1.000 | 1.000 |
| CRP >80 mg/dl | 0.004 | 2.612 | 1.643 | 4.921 |
| Bacteremia | 0.603 | 1.530 | 0.309 | 7.575 |
| Non-E.coli infection | 0.228 | 2.345 | 0.587 | 9.368 |
| Acute Kidney Injury | 0.025 | 2.307 | 1.149 | 4.902 |
| Constant | 0.308 | 0.000 |  |  |

Note: The dependent variable in the logistic regression model is the presence of urinary tract malformation in the imaging test (vs. absence of imaging abnormalities). The table presents the independent variables included in the analysis and their corresponding statistical associations with urinary tract malformation expressed as odds ratios [Exp(B)] with 95% confidence intervals (CI) and significance value (Sig.). CRP: C-Reactive protein.
